# Personal and Social Transformative Learning through Community‑Based Education: Insights from Training Socially Accountable Medical Doctors at a Historically Disadvantaged University in the Eastern Cape, South Africa

**DOI:** 10.5334/aogh.4560

**Published:** 2024-11-28

**Authors:** Siyonela Mlonyeni, Sibusiso Cyprian Nomatshila, O.R Mnyaka, Laston Gonah, Olanrewaju Oladimeji

**Affiliations:** 1 Department of Public Health, Faculty of Health Sciences, Walter Sisulu University, Mthatha 5100, Eastern Cape, South Africa; 2 Department of Public Health, School of Healthcare Sciences, Sefako Makgatho Health Sciences University, Ga‑Rankuwa, Pretoria 0208, South Africa

**Keywords:** Personal transformation, social transformation, transformative learning, integrated longitudinal clerkship, community‑based education

## Abstract

*Background:* In 2014, the Faculty of Health Sciences at Walter Sisulu University introduced a 20‑week long integrated longitudinal clinical clerkship (ILCC) rotation block as part of its commitment to community‑based education and social responsiveness, with the goal of ensuring that the curriculum is updated to align with the contemporary health system challenges in South Africa.

*Aim:* To explore whether medical student participants underwent social and personal transformative learning in understanding complex societal health needs during their integrated longitudinal community clerkship program.

*Methods:* This was an exploratory qualitative research study conducted among 113 5^th^ year medical students based at 8 selected hospitals during their 20‑week‑long community clerkship. Data were collected through six focus group discussions, complemented by data from reflective learning journal entries. Audio recordings were transcribed verbatim and merged with complementary data for thematic analysis in NVivo Version 13®.

*Results:* Adaptation challenges, improved social relations, coping with work demands, acquisition of relevant knowledge and skills, perceived inadequate support from the training institution and perceived lengthy programme duration emerged as key themes and were linked to personal and social transformation.

*Conclusion:* Personal and social transformation may have transpired amongst the student participants, as demonstrated by the observed thematic consistency between data sources. Further complementary studies are required to assess whether there was a shift in students’ understanding of community health needs and how the ILCC may have assisted the students in responding to community needs to have a comprehensive conclusion on whether the ILCC can be a tool for transformative learning.

## Introduction

Contemporary world‑wide disparities in health outcomes and disease prevalence irrefutably demonstrate the existence of a gap between governance priorities/capacity and community health needs. Societal health needs and expectations have been known to far exceed health system capacity and presumed available resources [1]. These societal health needs are also known to be dynamic, uncertain and complex in nature, due to the continuously changing environmental circumstances. Therefore, the medical curriculum needs to stay relevant in addressing the everchanging health system‑related needs and challenges. Aligning students’ learning needs with community needs and government priorities is critical in addressing health system disparities. Medical schools are now faced with the responsibility to transform their existing curriculum and align it with the country’s health system needs and challenges [2, 3].

The social obligations of medical schools can be graded into three levels: social responsibility, social responsiveness and social accountability [4]. Social responsibility describes an institution’s understanding of the social mission. Socially responsible institutions acknowledge that they have an obligation to meet the needs of society [5, 6]. At the level of social responsiveness, schools conduct activities that respond to the priority health needs of the society by directing education, research and service activities. Socially responsive schools are identified with students who learn within the community and observe or participate in health‑related activities [5]. Social accountability is the highest level, encompassing all levels of social obligations. Socially accountable medical schools go beyond responding to needs and foresee the health needs of society and work with the community and key stakeholders to tailor training programmes [7].

Medical and health sciences schools reflect this by including public health policy, social determinants of health and community‑based education programmes in their curricula [4, 5]. Community engagement experiences can be a valuable pedagogical tool in offering students knowledge that may foster a better understanding of societal needs and professional accountability [8]. These experiences empower the students with a rich and transformative learning opportunity as they endeavour to understand and figure out the complexity of their own values and beliefs when immersed in the community [9].

The Faculty of Health Science at Walter Sisulu University (WSU) is one of the leaders in community‑based education (CBE) in South Africa [10]. In June 2014, the Faculty of Health Sciences at WSU introduced a compulsory 20‑week‑long extensive integrated longitudinal clinical clerkship (ILCC) rotation block for medical students, as part of its commitment to CBE, problem‑based learning (PBL) and social responsiveness [11].

The ILCC aims to empower students to fundamentally change their perspectives, behaviours and understanding of the world around them, from which their patients come and from which health problems emanate. The training programme seeks to produce medical doctors who can cope and relish the demands of working in a district hospital, being sensitive to community needs and practice their work in a manner that recognizes and meets these needs. As a tool for transformative learning, the implementation of the ILCC places emphasis on student–student peer interactions and critical reflective thinking on assigned practical learning activities; effective student mentorship by faculty and senior professionals at host district hospitals; fostering positive student–faculty relationships through continuous follow‑up and feedback mechanisms for troubleshooting; and undertaking monitoring and evaluation on the effectiveness of the ILCC programme through research.

Guided by the transformation learning framework outlined by Scott in their study on transformative learning conducted in Canada, the study sought to explore whether medical student participants had undergone social and personal transformative learning, including understanding of community needs, post the 20‑week‑long community clerkship [12]. The remaining two phases of students’ transformative learning, namely a shift in understanding of community needs and response to community needs, will be assessed and discussed in subsequent publications.

In accordance with ILCC goals, the transformation learning theory targets recognizing the processes and experiences that one or even a group must embark on to warrant that transformation has taken place [13]. According to Soctt’s findings from the Canadian study on adult education, the following set of criteria was postulated to determine transformative learning:

1. There must be structural change, either social structural transformation or personal transformation or both, including the creation of a disorientated dilemma, representing the initiation of a transformative learning experience characterised by life crises that trigger one to question their assumptions, and thereby resulting in transformed beliefs.
2. There must be a shift in what counts as knowledge.
3. Transformation is based on conflict theory, which assumes that there are always different interests within social groups and acquisition of knowledge and skills for implementing one’s plan [12].

## Methodology

### Study design

This was a cross‑sectional, exploratory study conducted using qualitative research methods. Data were collected from reflective learning journals documented by students throughout the 20‑week period and focus group discussions (FGDs) conducted on week 19 of the clerkship, using a pre‑tested and revised FGD guide.

### Data sources

Data were largely obtained from the independent researcher‑based analysis of 2 main data sources, namely: (1) student learning journals, which enable assessment of critical reflective thinking by the students. The student learning journal represents a key academic and examinable tool for assessing transformative thinking in line with set learning objectives. These learning journals include prospectively conducted, real dialogue amongst multiple learners concerning how they would have understood and achieved the set learning objectives and allowing for mentors or instructors to confirm whether the required competences would have been acquired by the students; and (2) the yields from focus group discussions (FGDs) with students, which covered questions requiring reflective thinking according to set ILCC objectives, involving student self‑reflections on their opinions and experiences. The consistency in data identified between these two independently conducted methods served as the basis for measuring transformation.

### Study setting

The study was conducted at 8 hospitals selected from 5 districts in the Eastern Cape, namely: (1) All Saints Hospital, Engcobo, (2) Dr Malizo Mpehle Memorial Hospital, Tsolo, (3) Frontier Hospital, Queenstown, (5) Madzikane KaZulu Memorial Hospital, Mount Frere, (6) Settlers Hospital, Grahamstown, (7) St Elizabeth Hospital, Lusikisiki, and (8) St Patricks Hospital, Mbizana. These hospitals are situated in the rural or the semi‑rural parts of the Eastern Cape, with each hospital having a unique population and catchment area to serve. The students were allocated to these 8 hospitals, where they completed their ILCC programme.

### Population and sampling

The ILCC programme is only meant for 5^th^ year medical students. With a total number of 113 medical students registered for their 5^th^ year in 2021, the total population sampling technique was applied where every 5^th^ year medical student participated.

### Data collection

Data was collected from all 5^th^‑year medical students based at the 8 selected study sites, where the students were attached for the 20‑week‑long community clerkship programme.

Data from FGDs was augmented with field notes, session summaries, debriefing notes and observation techniques, in addition to data from reflective learning journal entries. Reflective learning journal entries contained rich information on students’ field experiences, student–student peer interactions and critical reflective thinking based on conducted learning tasks. In this study, reflective journaling means relating a recent experience and unpacking striking observations (e.g. people, resources, activities) that affected or influenced learning. A pre‑tested and revised FGD guide was used to conduct the FGDs. A total of six (6) FGDs were conducted, with each consisting of 8–12 participants and lasting for not more than 60 minutes. Observation notes were taken during the FGDs to aid the researcher in observing and positioning the epistemology on the students’ understanding of community needs. Probing was done throughout the discussions to stimulate critical thinking for achieving data clarity and extracting rich information on participants’ personal opinions, feelings and experiences.

### Data management and analysis

The data from the FGDs were stored as audio recordings, FGD notes, observation and session summaries, and journal summaries. All audio files were transcribed verbatim onto a word‑processing programme, following which the audio‑recordings were transferred to a password‑protected cloud‑based storage system for safekeeping. All data collected using hard copies were scanned and stored in a password‑protected cloud‑based storage system for safekeeping.

To improve rigour, all transcripts were verified by an independent researcher using the audio recordings and original journal entries. The researchers also read all the transcripts to familiarize themselves with the data for analysis and for validation.

Analyst triangulation was achieved through two qualitative research experts independently analysing emerging codes and themes from the data sets yielded by FGDs and reflective learning journals, and later discussing them together to achieve results grounded on data rather than on personal perceptions. Thematic analysis was done in NVivo version 13® to identify different themes emerging from the data. Findings were structured according to the identified themes, complemented by verbatim quotes from the student participants to illustrate and support the analysis.

## Results

**Figure 1:**
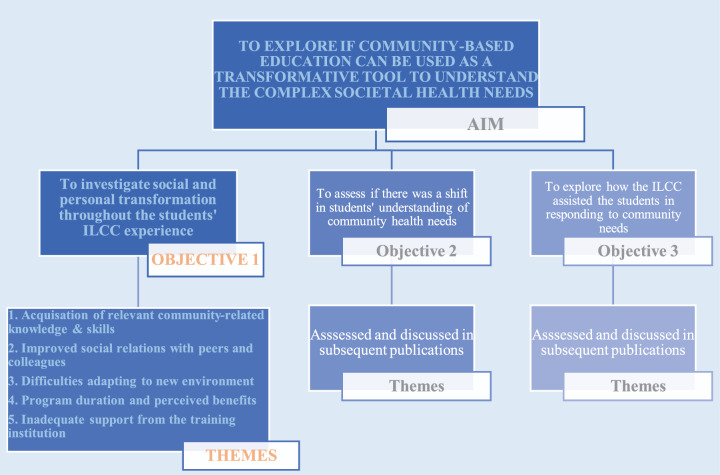
Results tree diagram.

The study investigated whether the students had undergone any social or personal transformation during the ILCC rotation. Five themes emerged from the data, as shown in Figure 1 and Table 1.

**Table 1:** Saturation grid of themes emerging from FGDs and reflective learning journals.

THEMES	FOCUS GROUP DISCUSSION/JOURNAL ENTRY NUMBER							
**Focus group discussions (FGDs)**	**1**	**2**	**3**	**4**	**5**	**6**		
1. Acquisition of relevant community‑related knowledge and skills	X	X	X	X	X	X		
2. Improved social relations with peers and colleagues	X	X	X	X	X	X		
3. Difficulties adapting to a new environment	X	X	X	X	X	X		
4. Programme duration and perceived benefits		X		X	X			
5. Inadequate support from the training institution	X		X	X				
**Reflective journal entries**	**1**	**2**	**3**	**4**	**5**	**6**	**7**	**8**
1. Acquisition of relevant community‑related knowledge and skills	X	X	X	X	X	X	X	X
2. Improved social relations with peers and colleagues	X	X	X	X	X	X	X	X
3. Difficulties adapting to a new environment	X	X		X	X	X	X	
4. Programme duration and perceived benefits	X	X			X	X		
5. Inadequate support from the training institution	X			X	X			

**1. Acquisition of relevant community‑related knowledge and skills**

There was convergence of views from all FGD responses and reflective learning journals that being placed at district level through the ILCC allowed the students to experience community social influence, closely understand community structure and function, familiarize themselves with the social determinants of health, and learn more about patient care. The students concurred that this community‑based learning exposure was useful in making them sensitive to community needs and work in a manner that recognizes and meets those needs. The ILCC gave the students an opportunity to experience the provision of continuous care for the patients and appreciate the practical operation and benefits of the referral system. According to most emerging themes, the placement helped the student participants to be closer to their patients and taught them to do better patient follow‑up, thereby promoting personal and professional growth:

*“ I am now well informed when I must refer a patient because I have learnt how to refer a patient, how to book patients, and how to follow up a patient when the patient comes back, and when we have discharged them. Through this program, I learnt that patient care doesn’t end with just admitting the patient, treating them and discharging them. How you also understand the conditions in which they live and follow‑up that patient matters too.”* (Male, Journal Entry 7, Hospital 7 Student)

*“One more thing that I think I’ve appreciated a little bit more here is the opportunity I got for hands‑on practice on the patients and the community. … because in tertiary hospitals there are so many more senior doctors who are higher up the hierarchy than me, we just go there to observe and not to practice, you look it up”* (Male, Journal Entry 5, Hospital 7 Student)

**2. Improved social relations with peers and colleagues**

There was commonality of findings from the gathered data confirming that being placed in a facility with people from diverse professional and cultural backgrounds, and having different views and thoughts about issues were important highlights for the student participants. Amongst the most common themes to emerge from the data was that the rotation allowed the students to embrace the various group dynamics within their teams, including accumulation of some team building and conflict resolution skills, being able to unify the working strategies to work well with each other and recognize new roles and relationships. They learnt how rural doctors cared for their patients, how they diligently carried out their duties and how the students were able to form positive relations with the various health care providers:

*“Although there was initially that clash among us as we came together trying to know each other’s personalities, we eventually managed to understand each other better. Despite the initial conflicts, we ended up working more effectively together, respecting, acknowledging and supporting each other better than before. So, I think for us the ILCC is a very, very great learning opportunity not to be missed,”* (Female, Journal Entry 2, Hospital 3 Student)

**3. Difficulties adapting to new environment**

Student participants highlighted several challenges they had encountered at the beginning of, and throughout the ILCC block rotation, with marked consistencies within and between datasets. It emerged that starting the ILCC block rotation was associated with confusion and uncertainty, as the students were not quite sure about what they were expected to do and were anxious about the possibility of failing on rotation assessments. Common themes pointed to shared sentiments about how overwhelming it felt relocating from the more urban university setting to a rural setting. Upon arrival at their allocated placement sites, adjusting to a life in the rural or peri‑urban setting proved to be a challenge, especially due to non‑availability of certain essential personal items/supplies, and the associated long distances they had to travel to acquire the items.

The students consistently highlighted that the new environment made them feel isolated, as they felt separated from the people and environment they were used to. However, there was a general concurrence amongst FGD responses and reflective learning journal data, which emphasized that with time all the students successfully adapted to their respective new environments:

*“It was very difficult to cope with the demands of working in a remote setting that I didn’t know …. I was always afraid that I will fail this whole thing. I didn’t know anyone even the senior doctors; I didn’t know where to get essential personal items; and I wasn’t comfortable talking to people here…... When I arrived here, I felt the programme wasn’t working very well for me until when I was about half‑way through, that is when I became comfortable with the environment and people around me. Eventually, I was free to ask questions from other healthcare workers here. Looking back, coping was difficulty but necessary – I appreciate the opportunity of having been here. Who knows, maybe one day I will work in a remote place like this.* (Male, Journal Entry 5, Hospital 1 Student)

**4. Programme duration and perceived benefits**

There was a convergence of views from some FGDs and journals that, despite some benefits and learning opportunities created by the ILCC rotation, the duration of programme was considered too lengthy for the student participants:

*“Honestly…. initially, I was like why five months? But now, I feel like it was shorter, you probably wouldn’t have gotten that much experience.”* (Female, Journal Entry 8, Hospital 6 Student)

*“To me it’s not a waste of time because I have gained experience and some exposure to primary health care and undertaking social diagnosis. I am now able to think critically and be responsible in dealing with some clinical situations, without much handholding or hesitancy. In fact, I can now confidently conduct patient consultations including history taking to assess, diagnose and manage some cases. It’s not a complete waste of time, for sure. But a programme that lasts five months?... I mean, it’s too long.”* (Male, Journal Entry 2, Hospital 2 Student)

**5. Inadequate support from the training institution**

Inadequate support from the training institution emerged as one of the commonly felt challenges faced by student participants. The students felt that the University sent them to these remote facilities without providing adequate preparation, follow‑up support and supervision. FGD responses and journal entries consistently pointed to feelings of frustration about being allocated to remote hospitals, which they initially believed could not help them to acquire new relevant knowledge.

*“At first, I felt like this University just took us and left us here and like, see you in five months. The program is good, but the University can do more like communicating and checking on us from time to time.”* (Male, Journal Entry 1, Hospital 3 Student)

The frustrated participants further expressed how they prefer learning in the traditional teaching hospital environment as compared with the rural hospitals.

*“I don’t think I have learnt anything special from this remote place. In fact, even I have receded in knowledge in this time that I have been moved.”* (Male, Journal Entry 9, Hospital 4 Student)

## Discussion

The study investigated whether students had undergone social and personal transformative learning in understanding complex societal health needs during their integrated longitudinal community clerkship from the perspectives and experiences of medical students participating in the ILCC programme. Key themes of relevance to the study aim include:

(1) acquisition of relevant community‑related knowledge and skills, (2) improved social relations with peers and colleagues, (3) difficulties adapting to a new environment, (4) programme duration and perceived benefits, and (5) inadequate support from the training institution.

The study established a successful coping mechanism over time. Generalized difficulties adapting to new environments and improved social relations with peers and colleagues were some of the key themes that emerged from the study. The narrations and personal reflections consistently demonstrated that the participants initially experienced dilemma, difficulties and some discomforts in adjusting to life in rural settings for the ILCC programme, in addition to perceived lengthy program duration and perceived inadequate support from the training institution. Herbers defines a disorienting dilemma as a catalyst for change in a perspective that may culminate in transformative learning. He further explains that a disorienting dilemma can prompt students and faculty to reflect on assumptions about self and society, leading to deeper self‑understanding and increased awareness of flawed assumptions reinforced by one’s culture [14]. There was a consensus that the students managed to successfully cope with and relish the demands of working in a district hospital. Mezirow’s theory of perspective transformation offers a model for understanding and implementing the process of change [15]. The model captures how the setting shaped the scope and nature of student learning and socialization experience, both professionally and socially. The results of this research study suggest that the clerkship allowed the students an opportunity for sensemaking, as it emerged that they had the opportunity to interact with the community and reflect on their social background and cultural/religious beliefs. The common themes pointed to the role played by positive and negative experiences in shaping social and personal skills such as team building, conflict resolution and coping, thereby contributing to personal and professional growth. As demonstrated by study findings, the ILCC programme successfully equipped the students with practical knowledge and skills on relevant aspects of community health, such as patient care, primary health care, healthcare delivery system, community diagnosis and social determinants of health, as required.

Assessment of students’ reflective learning journals compared with the ILCC programme goals demonstrated that the students had successfully acquired clinical‑/community‑related knowledge and skills on social determinants of health, community diagnosis and patient care. This was consistent with FGD data findings, as the FGDs were characterized by open discussion and critical reflective thinking, with the participants demonstrating a shift in knowledge, and attainment of ILCC learning outcomes.

Overall, study findings suggest that some personal and social transformation may have transpired amongst the student participants, as demonstrated by the observed thematic consistency between data sources. The study relied on use of qualitative techniques to measure transformative learning and could not quantify the magnitude of the transformation. We, however, believe that the methodological strength from the use of the examinable reflective learning journals and FGDs as data sources should be considered a merit, and far exceeds exclusive reliance on self‑assessment alone. Scales for measuring transformative learning quantitatively have been proposed, but they are still in their formative stages, requiring rigorous validation, which was beyond the scope of an exploratory study. This study may be used as a springboard for future scale‑based quantitative research studies on measuring transformative learning.

### Limitations of the study

- Social and personal knowledge and skills compared with ILCC learning objectives was not determined at baseline, that is, before commencement of the ILCC rotation. Though the study made use of retrospective assessments to estimate the baseline perceptions, retrospective assessments are known to be prone to recall bias. Personal and social transformative learning could have been better assessed by making comparison between baseline and end‑of‑programme assessment scores.
- Key informant interviews were not conducted with mentors at host attachment sites or facilitators at the training institution. These interviews would have complemented the data on social and personal transformative learning amongst students. This missed opportunity could have provided useful complementary information and improved the quality of study findings.
- Transformative learning was not determined quantitatively. However, the development of quantitative scales and validating them to measure transformation was beyond the scope of an exploratory study.

## Conclusion

Personal and social transformation may have transpired amongst the student participants, as demonstrated by the observed thematic consistency between data sources. Further complementary studies are required that will assess whether there was a shift in students’ understanding of community health needs and how the ILCC assisted the students in responding to community needs to have a comprehensive conclusion on whether the ILCC can be a tool for transformative learning.

## Data Availability

The datasets used and/or analysed during the current study are available from the corresponding author on reasonable request.
